# Diagnostic model based on key autophagy-related genes in intervertebral disc degeneration

**DOI:** 10.1186/s12891-023-06886-w

**Published:** 2023-12-01

**Authors:** Yifeng Wang, Zhiwei Wang, Yifan Tang, Yong Chen, Chuanyuan Fang, Zhihui Li, Genlong Jiao, Xiongsheng Chen

**Affiliations:** 1 Department of Spine Surgery, The First Affiliated Hospital, Jinan University, Guangzhou, Guangdong 510630 P.R. China; 2 Department of Orthopaedic Surgery, The First Affiliated Hospital of Xiamen University, Xiamen, Fujian 361003 P.R. China; 3 Spine Center, Department of Orthopaedics, Changzheng Hospital, Naval Medical University(Second Military Medical University, 415 Fengyang Road, Shanghai, 200003 P.R. China; 4 Department of Spine Surgery, The Sixth Affiliated Hospital, Jinan University, Dongguan, Guangdong 523570 P.R. China; 5 Department of Orthopaedics, Shanghai General Hospital, Shanghai Jiao Tong University School of Medicine, Shanghai, 20008 P.R. China

**Keywords:** Intervertebral disc degeneration, Autophagy, Diagnostic model, Immune cells infiltration

## Abstract

**Background:**

Current research on autophagy is mainly focused on intervertebral disc tissues and cells, while there is few on human peripheral blood sample. therefore, this study constructed a diagnostic model to identify autophagy-related markers of intervertebral disc degeneration (IVDD).

**Methods:**

GSE150408 and GSE124272 datasets were acquired from the Gene Expression Omnibus database, and differential expression analysis was performed. The IVDD-autophagy genes were obtained using Weighted Gene Coexpression Network Analysis, and a diagnostic model was constructed and validated, followed by Gene Set Variation Analysis (GSVA) and Gene Set Enrichment Analysis (GSEA). Meanwhile, miRNA–gene and transcription factor–gene interaction networks were constructed. In addition, drug-gene interactions and target genes of methylprednisolone and glucosamine were analyzed.

**Results:**

A total of 1,776 differentially expressed genes were identified between IVDD and control samples, and the composition of the four immune cell types was significantly different between the IVDD and control samples. The Meturquoise and Mebrown modules were significantly related to immune cells, with significant differences between the control and IVDD samples. A diagnostic model was constructed using five key IVDD-autophagy genes. The area under the curve values of the model in the training and validation datasets were 0.907 and 0.984, respectively. The enrichment scores of the two pathways were significantly different between the IVDD and healthy groups. Eight pathways in the IVDD and healthy groups had significant differences. A total of 16 miRNAs and 3 transcription factors were predicted to be of great value. In total, 84 significantly related drugs were screened for five key IVDD-autophagy genes in the diagnostic model, and three common autophagy-related target genes of methylprednisolone and glucosamine were predicted.

**Conclusion:**

This study constructs a reliable autophagy-related diagnostic model that is strongly related to the immune microenvironment of IVD. Autophagy-related genes, including *PHF23*, *RAB24*, *STAT3*, *TOMM5*, and *DNAJB9*, may participate in IVDD pathogenesis. In addition, methylprednisolone and glucosamine may exert therapeutic effects on IVDD by targeting *CTSD*, *VEGFA*, and *BAX* genes through apoptosis, as well as the sphingolipid and AGE-RAGE signaling pathways in diabetic complications.

## Highlights

The autophagy-related diagnostic model closely associated with immunity was constructed based on five key autophagy-related genes in IVDD patients’ peripheral blood sample, which could diagnose of IVDD.

Methylprednisolone and glucosamine, empirical used clinical therapeutic drugs for IVDD, might exert therapeutic effect by targeting *CTSD*, *VEGFA*, and *BAX* through apoptosis, sphingolipid signaling pathway, and AGE-RAGE signaling pathway in diabetic complications.

This study provides new insights for the diagnosis and therapy of IVDD.

## Background

The intervertebral disc (IVD), a soft tissue structure connects the upper and lower vertebral bodies, is composed of the nucleus pulposus, annulus fibrosus, and cartilage endplate [1, 2]. The functions of IVD include buffering pressure and maintaining stability, physiological curvature, and flexibility of the spine [2]. IVD degeneration (IVDD), a common chronic degenerative disease, is the leading cause of low back pain and seriously affects the health and quality of life of the aged [3, 4]. It is suggested that the prevalence rate of IVDD among middle-aged and older people can be above 90%; however, with the acceleration of population aging and changes in work and lifestyle, the incidence rate has been increasing annually, showing an increasing trend in the youth [5, 6]. The etiology of IVDD is far from being understood, however, there is consensus that not a single factor can be held responsible for the complex phenomenon of disc degeneration. Rather a multitude of exogenous and endogenous factors might influence the progress of degenerative changes of the discs. Insufficient nutritional supply of the disc is thought to be a primarily problem contributing to disc degeneration, while genetic predisposition also has a major impact on IVDD. Polymorphisms affect genes that are involved in the maintenance of integrity or functionality of the disc matrix, suggesting that the genetic background plays a major role in the integrity of a healthy disc [7]. Currently, many methods are used in the treatment of IVDD, including drug therapy, surgical treatment, and physical therapy; however, they only perform lenitive function and finally remove the disc [8–10]. Therefore, given the high incidence of IVDD and aggravation of the disease, there is an urgent need to identify effective diagnostic and treatment measures for patients.

Autophagy refers to a dynamic process of highly conserved intracellular and lysosome-dependent degradation [11, 12]. While intracellular autophagy remains at a relatively low level under normal circumstances, autophagy levels can be upregulated under the action of some drugs or environmental stress, such as amino acid depletion, oxidative stress, and hypoxia [13, 14]. Therefore, autophagy disorders are associated with various diseases, including cancers, cardiovascular diseases, and chronic infectious diseases [15–17]. In recent years, autophagy has been demonstrated to play a pivotal role in the process of IVDD [18, 19], and the regulation of autophagy could protect against and postpone the progress of IVDD [20, 21]. However, the key autophagy-related genes and molecular mechanisms involved in IVDD remain enigmatic.

**Fig. 1:**
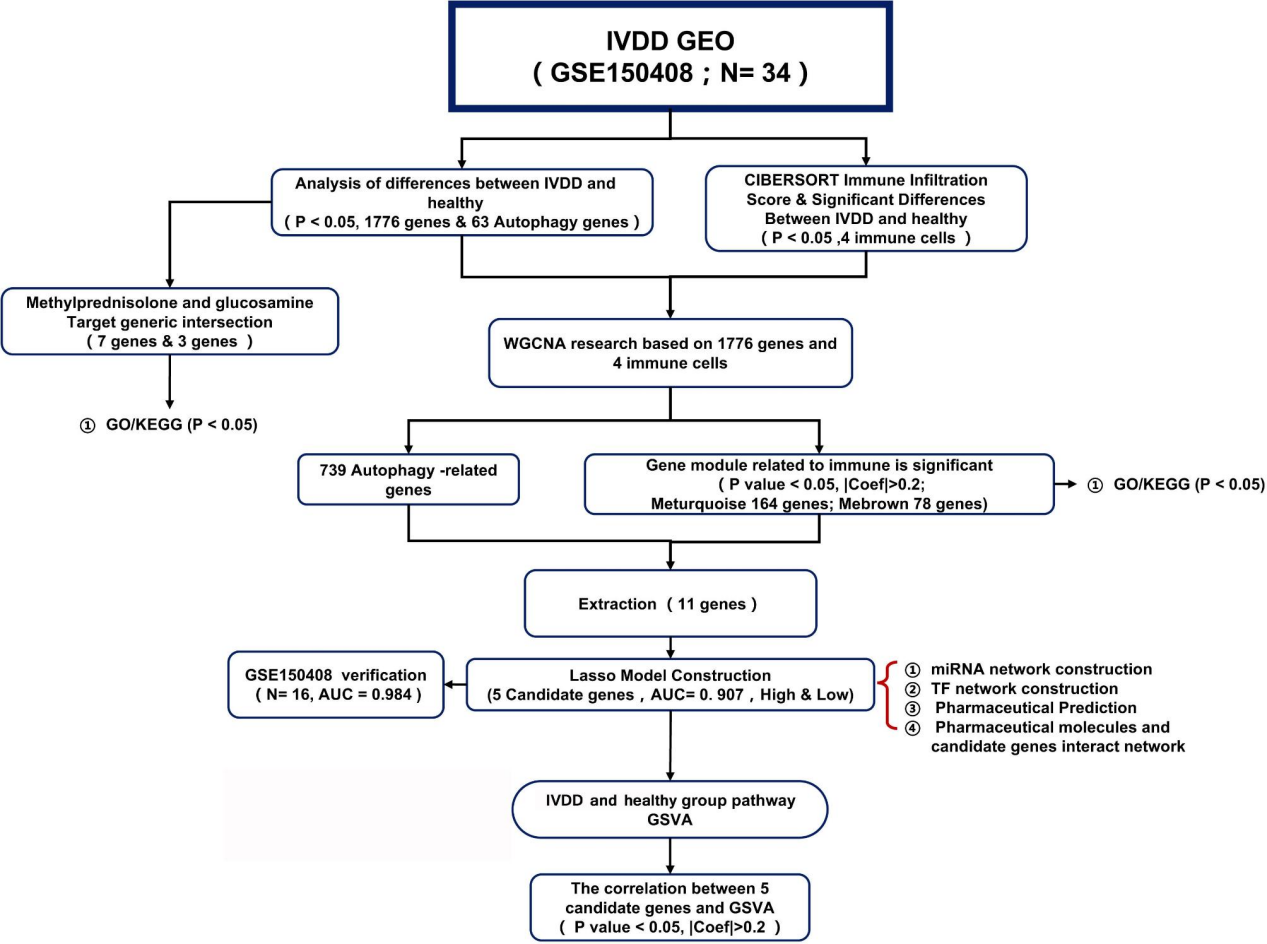
Schematic diagram of the study design

Currently, the early diagnosis of IVDD relies on time-consuming MRI scans, and the research on autophagy is mainly focused on IVD tissues and cells rather than human peripheral blood samples [19]. However, identifying key autophagy-related genes for IVDD could facilitate its early diagnosis, and the peripheral blood offers great advantage in view of the convenience of sampling. Fortunately, several studies have shown that the utilization of gene expression profiles and machine learning can serve to identify novel biomarkers in IVDD [22, 23]. Thus, this study aimed to identify autophagy-related markers and molecular mechanisms involved in IVDD and construct a diagnostic model based on human peripheral blood samples. This study provides novel and unique insights into IVDD treatments and therapeutic strategies. Figure 1 shows a schematic representation of the study design.

## Materials and methods

### Data collection and processing

Autophagy-related genes were acquired from the Human Autophagy Database and references (PMID32065482 [24] and PMID33392087 [25]), and overlapping genes were deleted. Two datasets related to IVDD, GSE150408 and GSE124272, were obtained from the Gene Expression Omnibus (GEO) database [26]. To ensure the accuracy of the results, GSE150408 with a relatively large sample size (containing 17 control samples and 17 IVDD samples) was used as a training dataset for diagnostic model construction, while GSE124272 (containing eight control samples and eight IVDD samples) was used as the validation dataset. The gene expression matrix probe value was used to log2 standardize the data quality control stage of the model, and the annotation file “GPL21185” provided internally by GEO was utilized to convert the probe to Gene Symbol for gene annotation. The average value was selected as the expression value when several probes matched a single-gene symbol.

### Differentially expressed genes (DEGs) between IVDD and control samples

The “limma” package [27] in R software was used to screen the DEGs between IVDD and control samples with threshold of P < 0.05. The screened DEGs were intersected with autophagy-related genes to obtain autophagy-DEGs.

### Immune infiltration analysis

The fraction of 22 immune cell infiltrations between IVDD and control samples was explored using CIBERSORT [28], and the difference in the fraction of 22 immune cell infiltrations between IVDD and control samples was compared using the Mann–Whitney U test with a cut-off value of P < 0.05.

### Weighted gene coexpression Network Analysis (WGCNA)

To explore the correlation between the DEGs and immune cells, WGCNA was performed. First, genes were screened using the “WGCNA” package (v 1.71) [29] to control the quality of gene expression values, mainly based on the top 75% genes with a median absolute deviation > 0.01. Then hclust clustering of sample populations was carried out using the “sampleTree” function. Then the coexpression network of all DEGs between IVDD and control samples was constructed, and the “pickSoftThreshold” function in “WGCNA” package was employed both to obtain the optimum “power” value (R-square > 0.85) and built the coexpression network (maxBlockSize = 5000). The edge properties of undirected networks were calculated as follows: ${Abs\left(Cor\right(genex,geney\left)\right)}^{power}$. The edge properties of a directed network were calculated as follows: ${(1+\frac{Cor(genex,geney)}{2})}^{power}$. The formula for calculating the edge properties of the sign hybrid was: $Cor{(genex,geney)^{power}}ifCor > 0\,else\,0$. The “plotDendroAndColors” was used to draw the hierarchical clustering of gene modules. Then Spearman correlation was performed between gene modules and immune cells to identify significant differences between control and IVDD samples at a threshold P < 0.05 and |Coeff| > 0.2 to consider the modules as IVDD-related modules.

### Diagnostic model construction

The genes in the IVDD-related modules were intersected with autophagy-related genes to obtain IVDD-autophagy genes. Then, the key IVDD-autophagy genes were acquired using the least absolute shrinkage and selection operator (LASSO) regression. Considering the two-classification characteristics of case control studies, the study used the “glmnet” function to set family = “binary” for fitting, and the feature of the model was calculated using the following formula: ${feature}_{sample}=\sum _{1}^{n}{Coef}_{i}*{x}_{i}$ (where feature represents the feature value of the sample in the model; Coef_i_ represents the regression coefficient of the gene in LASSO regression; and x_i_ represents the gene expression). The samples were categorized into IVDD and healthy groups based on the median cut-off value of the feature_sample_. The “roc” function in pROC package was utilized to calculate the area under the curve (AUC) value to analyze the prediction performance. The GSE124272 dataset was used to validate the proposed model.

### Gene Set Variation Analysis (GSVA) and Gene Set Enrichment Analysis (GSEA)

Seventeen immune-related pathways were obtained from the ImmPort database, and GSVA analysis was performed. In addition, using the hallmark gene sets, the GSEA and GSVA were conducted. In addition, to investigate the potential mechanism of genes in the diagnostic model, enrichment analysis was performed using the clusterProfiler package [30] with a threshold of P < 0.05 and enrichment factor > 1.5.

### Construction of miRNA-gene, transcription factor (TF)-gene interaction networks

The MiWalk database was used to screen for common miRNAs of genes in the diagnostic model, Jaspar database [31] was used to predict the TF of genes in the diagnostic model, and miRNA/TF-target gene networks were constructed using Cytoscape software.

### Drug-gene interaction

The genes in the diagnostic model served as promising targets for searching for drugs in the CLUE website with a threshold of P < 0.05 and z-score > 2. Then, the STITCH database was used to construct a drug-gene interaction network with the parameter of the minimum required interaction score: medium confidence (0.400).

### Target genes of methylprednisolone and glucosamine

Methylprednisolone and glucosamine are the empirical used clinical therapeutic drugs for IVDD. To explore the autophagy-related target genes of methylprednisolone and glucosamine, their target genes were predicted in Comparative Toxicogenomics Database (CTD) and then intersected with autophagy-related genes/DEGs, followed by enrichment analysis.

## Results

### DEGs in control and IVDD samples

**Fig. 2:**
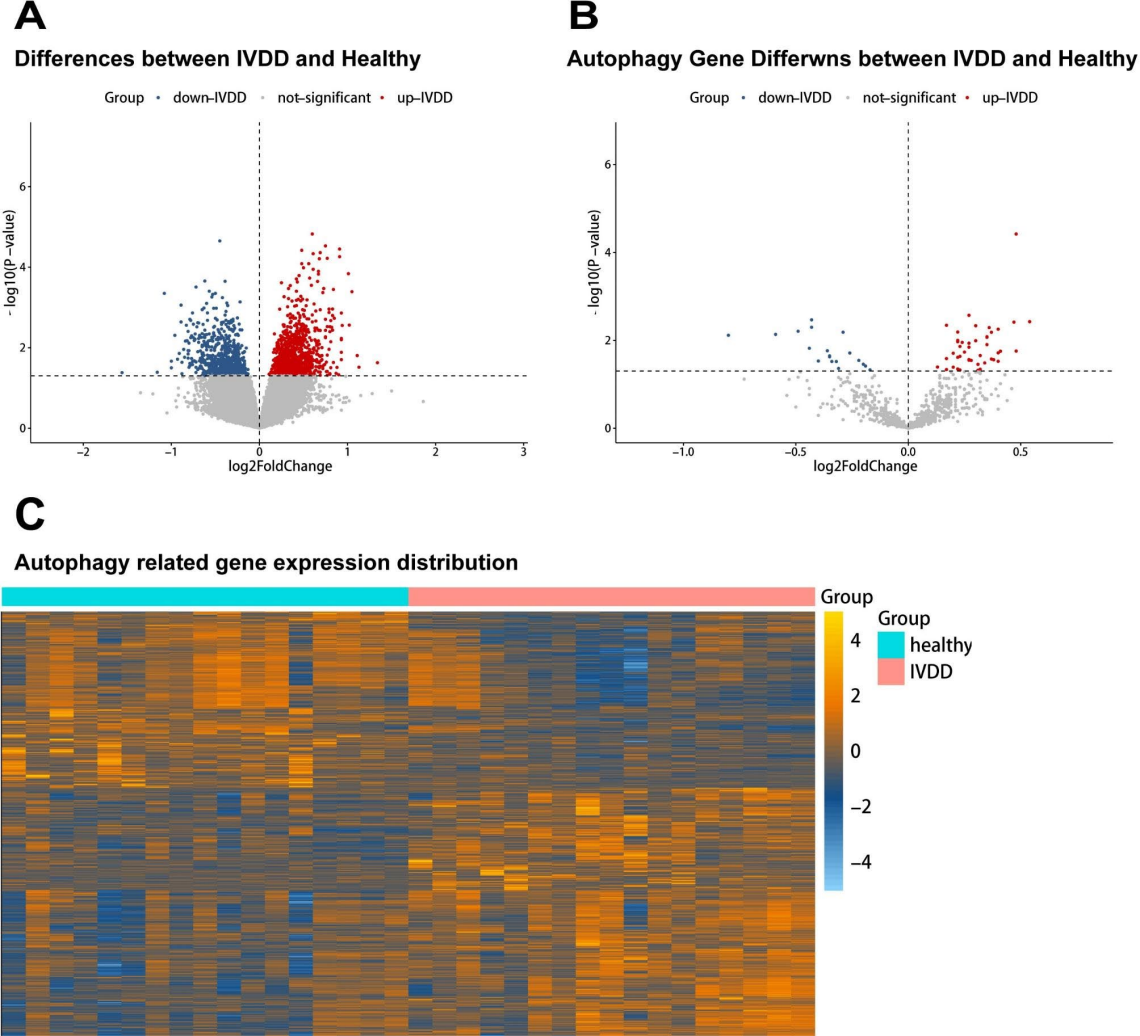
Differential expression analysis **(A)** Volcano plot of differentially expressed genes (DEGs) between intervertebral disc degeneration (IVDD) and control samples. Volcano plot **(B)** and heatmap **(C)** of autophagy-DEGs between IVDD and control samples

In total, 1,776 DEGs were screened between the control and IVDD samples (Fig. 2A), and 739 autophagy-related genes were identified. Then, the 1,776 DEGs were intersected with the 739 autophagy-related genes, and 63 autophagy-DEGs were obtained (Fig. 2B and C).

### Immune cells infiltration between control and IVDD samples

**Fig. 3:**
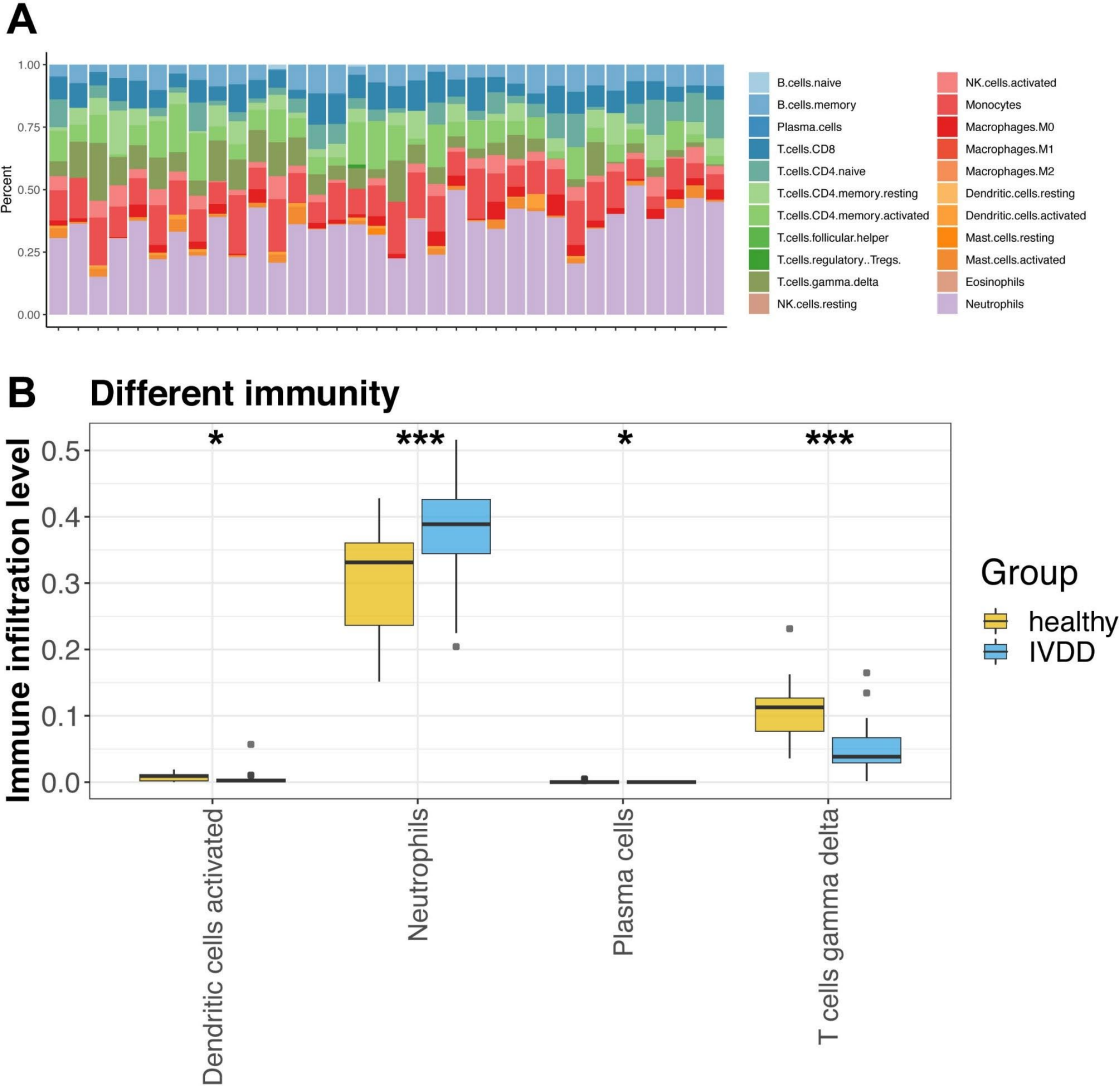
Immune infiltration analysis **(A)** The fraction of 22 immune cells infiltration between intervertebral disc degeneration (IVDD) and control samples **(B)** Four immune cells at infiltration level shows significant differences between control and IVDD samples. * P < 0.05, *** P < 0.001

The fraction of 22 immune cell infiltrates between IVDD and control samples were explored, and the fraction of neutrophils was found to be higher in IVDD samples than in control samples (Fig. 3A). In addition, as shown in Fig. 3B, activated dendritic cells, plasma cells, neutrophils, and gamma delta T cells differed significantly between the control and IVDD samples (P < 0.05).

### Construction of coexpression network and diagnostic model

**Fig. 4:**
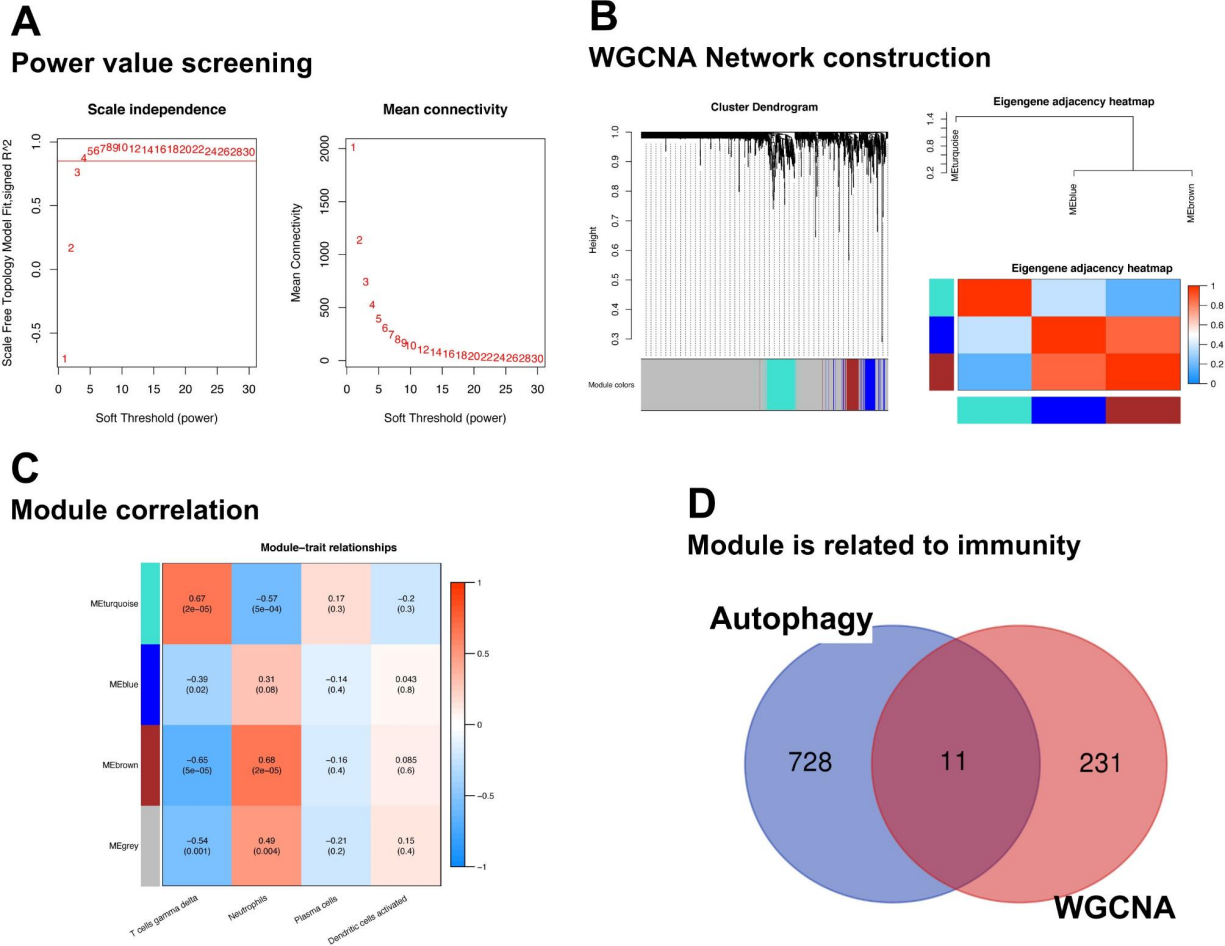
Weighted Gene Coexpression Network Analysis (WGCNA). **(A)** Hierarchical clustering of samples and selection of the weight parameter “power” of adjacency matrix and the mean connectivity **(B)** Tree diagram for module division **(C)** Global outline of the relationship between the modules and immune cells **(D)** Venn diagram of genes in meturquoise and mebrown modules and autophagy-related genes

**Fig. 5:**
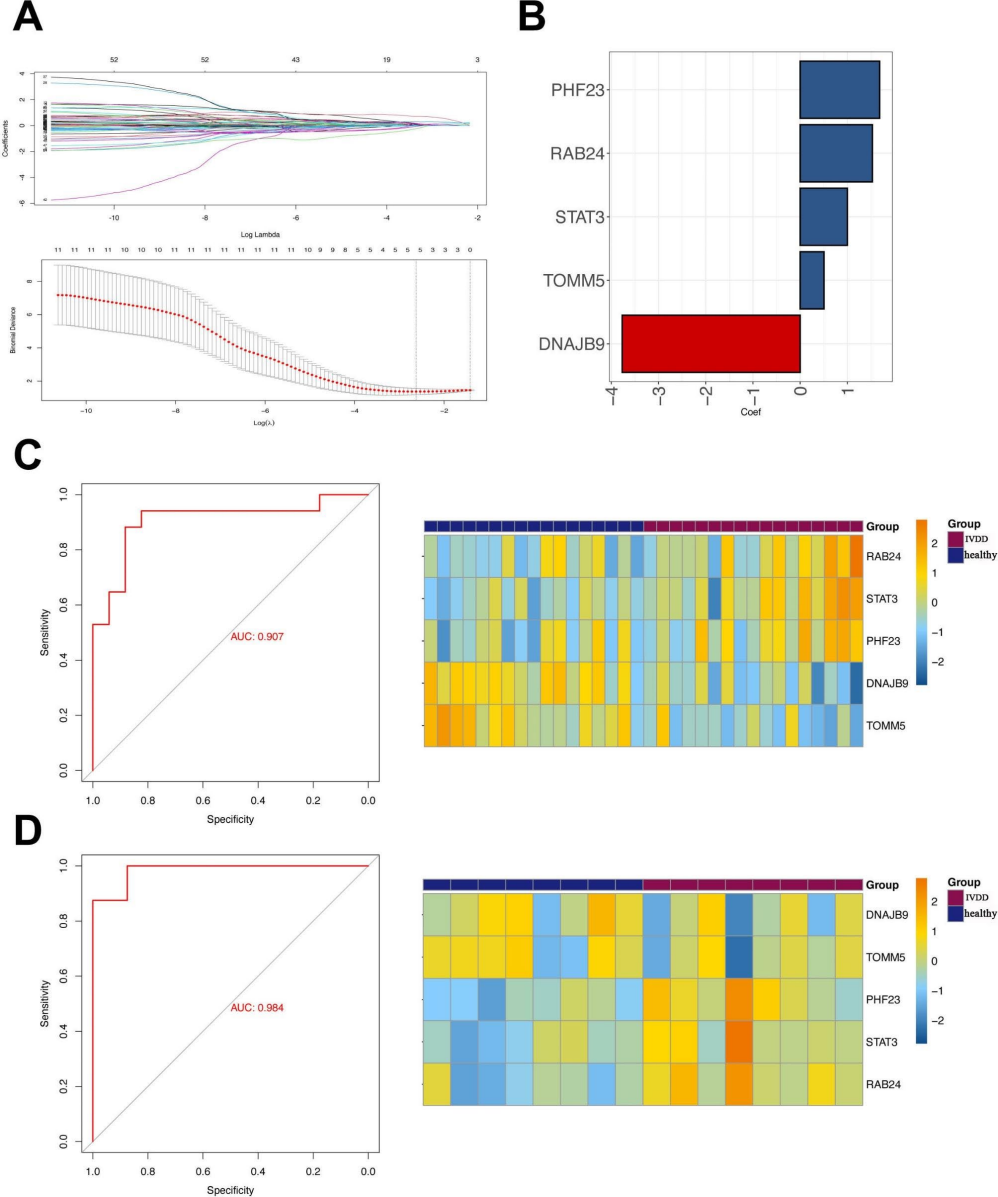
Construction of diagnostic model. **(A)** Least absolute shrinkage and selection operator (LASSO) regression. **(B) **Five key intervertebral disc degeneration (IVDD)-autophagy genes in diagnostic model. Receiver operating characteristic (ROC) curve of diagnostic model in training dataset **(C)** and validation dataset **(D)**

In total, 1,332 genes were screened to build the coexpression network, and the value of “power” (power = 4) when the R-square value reached 0.85 was selected (Fig. 4A). Three modules were obtained, including Meturquoise, Meblue, and Mebrown (Fig. 4B). Meturquoise and Mebrown modules were significantly related to immune cells with significant differences between control and IVDD samples and were considered as IVDD-related modules (Fig. 4C). The Meturquoise genes (164 genes) and Mebrown (78 genes) modules intersected with the 739 autophagy-related genes to obtain 11 genes, which were considered as IVDD-autophagy genes (Fig. 4D). Based on the 11 IVDD-autophagy genes, 5 key IVDD-autophagy genes were obtained using LASSO regression to construct the diagnostic model (Fig. 5A), including *PHF23, RAB24, STAT3, TOMM5*, and *DNAJB9* (Fig. 5B); and the AUC values of the model in the training and validation datasets were 0.907 and 0.984, respectively (Fig. 5C and D).

### GSVA and GSEA

**Fig. 6:**
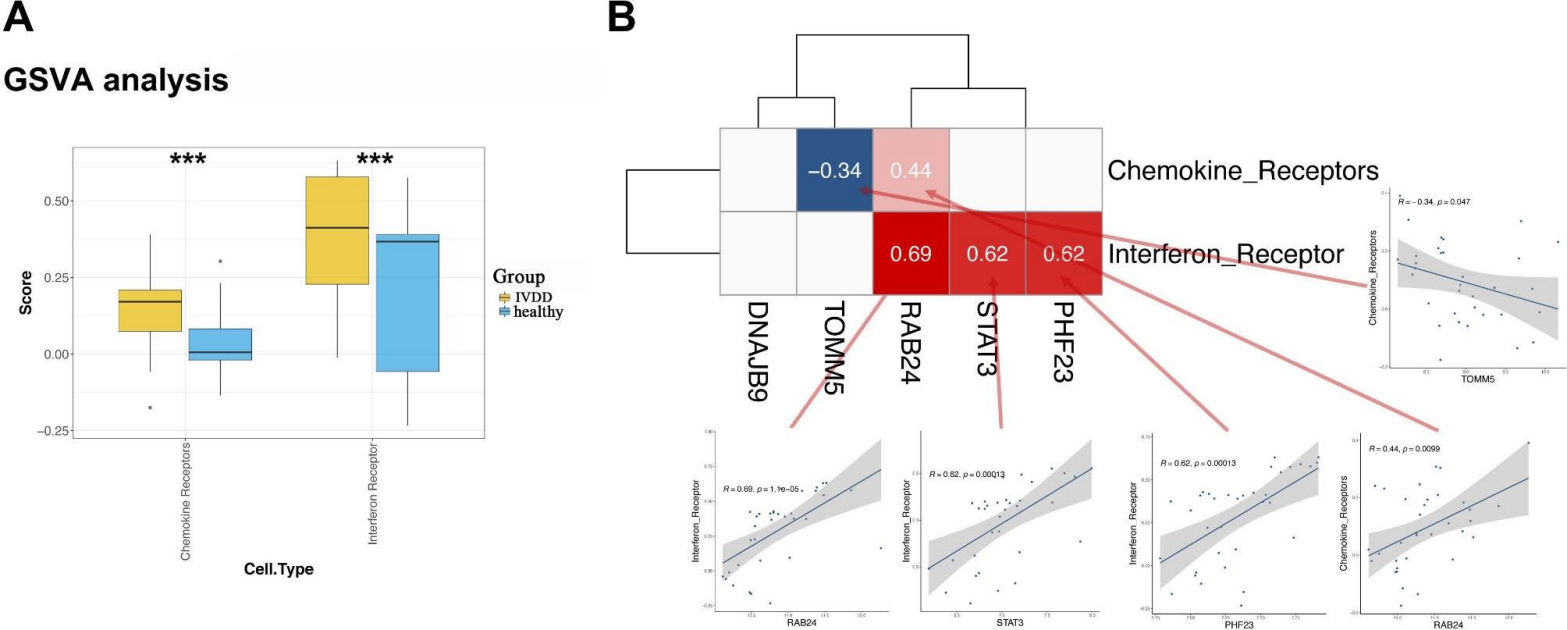
Gene Set Variation Analysis (GSVA) **(A)** The enrichment score of two pathways shows significant differences between intervertebral disc degeneration (IVDD) and healthy groups. *** P < 0.001. **(B)** Correlation analysis of gene expression matrix of diagnostic model and GSVA score of immune-related pathway

GSVA results showed that the enrichment scores of the two pathways showed significant differences between the IVDD and healthy groups, including interferon and chemokine receptors (Fig. 6A). The correlation analysis results showed that the five gene expressions of the five genes in the diagnostic model were significantly related with interferon and chemokine receptors, among which *RAB24* was the most significantly related with interferon receptor (Fig. 6B).

**Fig. 7:**
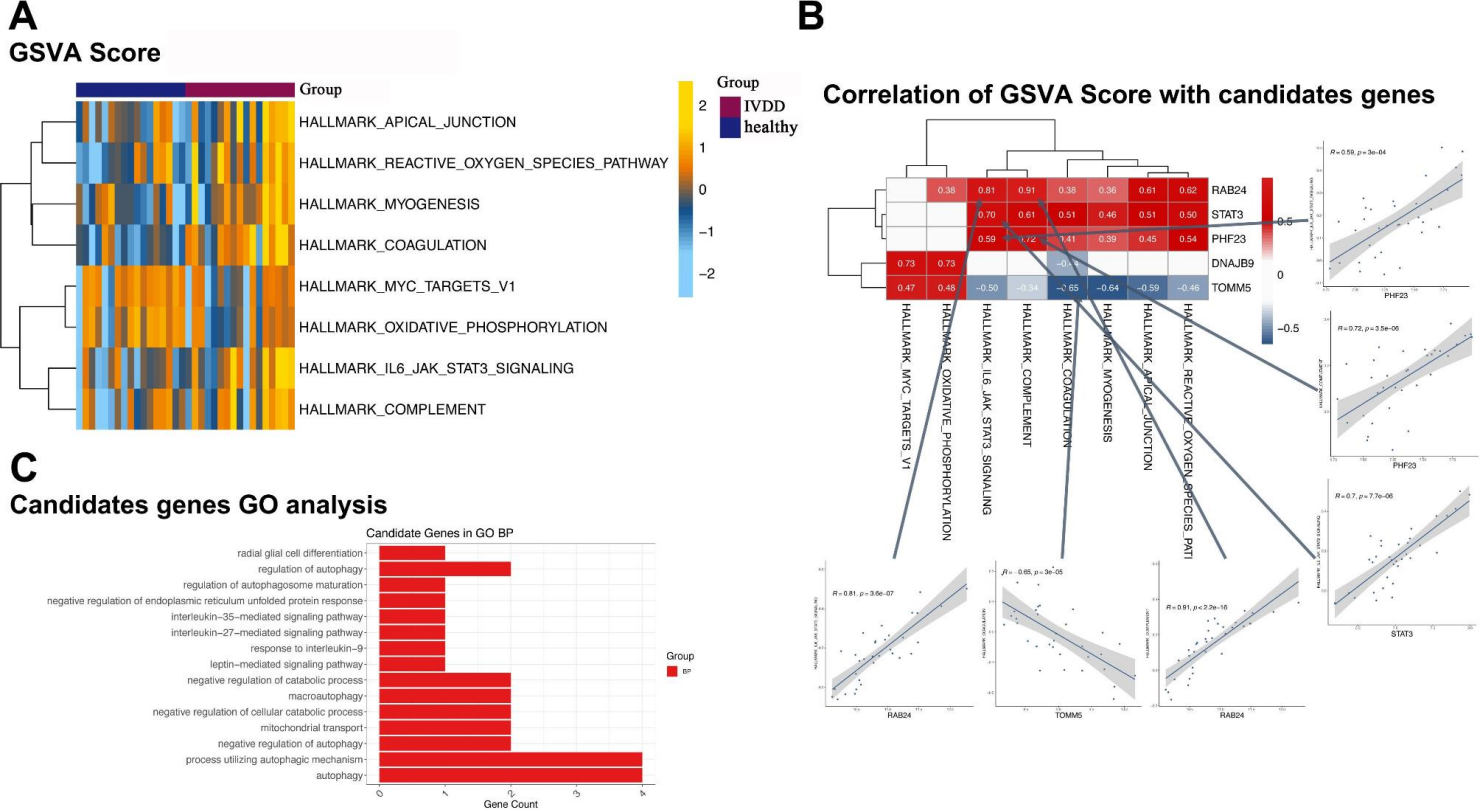
Gene Set Enrichment Analysis (GSEA). **(A)** Difference in GSVA score of hallmark geneset between intervertebral disc degeneration (IVDD) and healthy groups **(B)** Correlation analysis of gene expression matrix of diagnostic model and GSVA score of hallmark geneset **(C)** Enrichment analysis of genes in diagnostic model

As shown in Fig. 7A, eight pathways showed significant differences between IVDD and healthy groups. The correlation analysis results showed that the five gene expressions of the five genes in the diagnostic model was significantly related to the GSVA score of the eight pathways; among which, *RAB24* showed the most significant relation with the hallmark complement (Fig. 7B). Five genes in the diagnostic model showed significant enrichment in radial glial cell differentiation, regulation of autophagy, regulation of autophagosome maturation, and negative regulation of the endoplasmic reticulum unfolded protein response (Fig. 7C).

### MiRNA-gene, TF-gene interaction networks

**Fig. 8:**
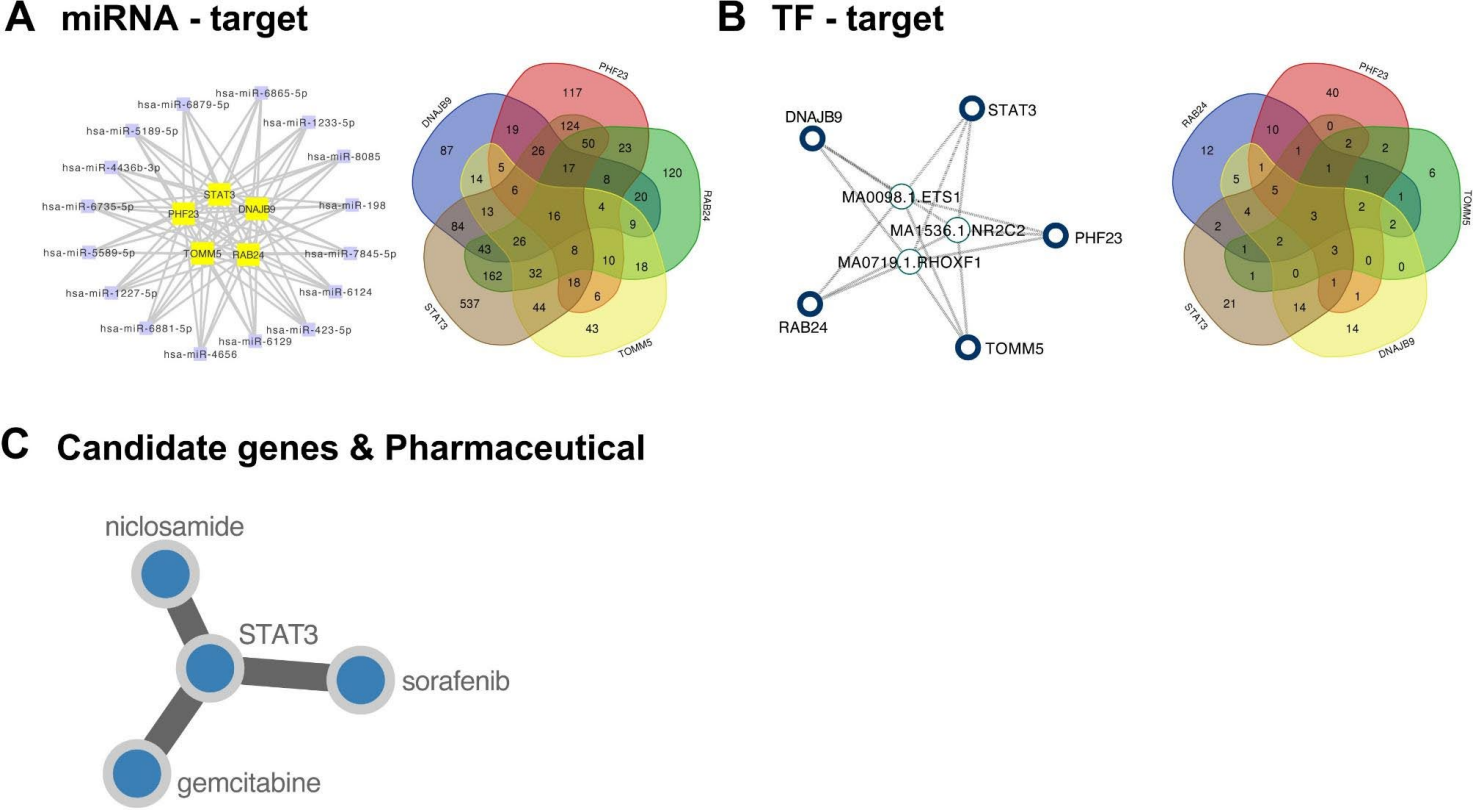
MiRNA-gene, transcription factor (TF)-gene interaction networks, and drug-gene interaction. **(A)** miRNA-gene interaction network **(B)** TF-gene interaction network **(C)** Drug-gene interactions

In total, 16 common miRNAs of five key IVDD-autophagy genes in the diagnostic model were screened in the MiWalk database, and three TFs of five key IVDD-autophagy genes in the diagnostic model were predicted in the Jaspar database. Then, the miRNA-gene and TF-gene interaction networks were constructed using Cytoscape software (Fig. 8A and B).

### Drug-gene interaction and target genes of methylprednisolone and glucosamine

**Fig. 9:**
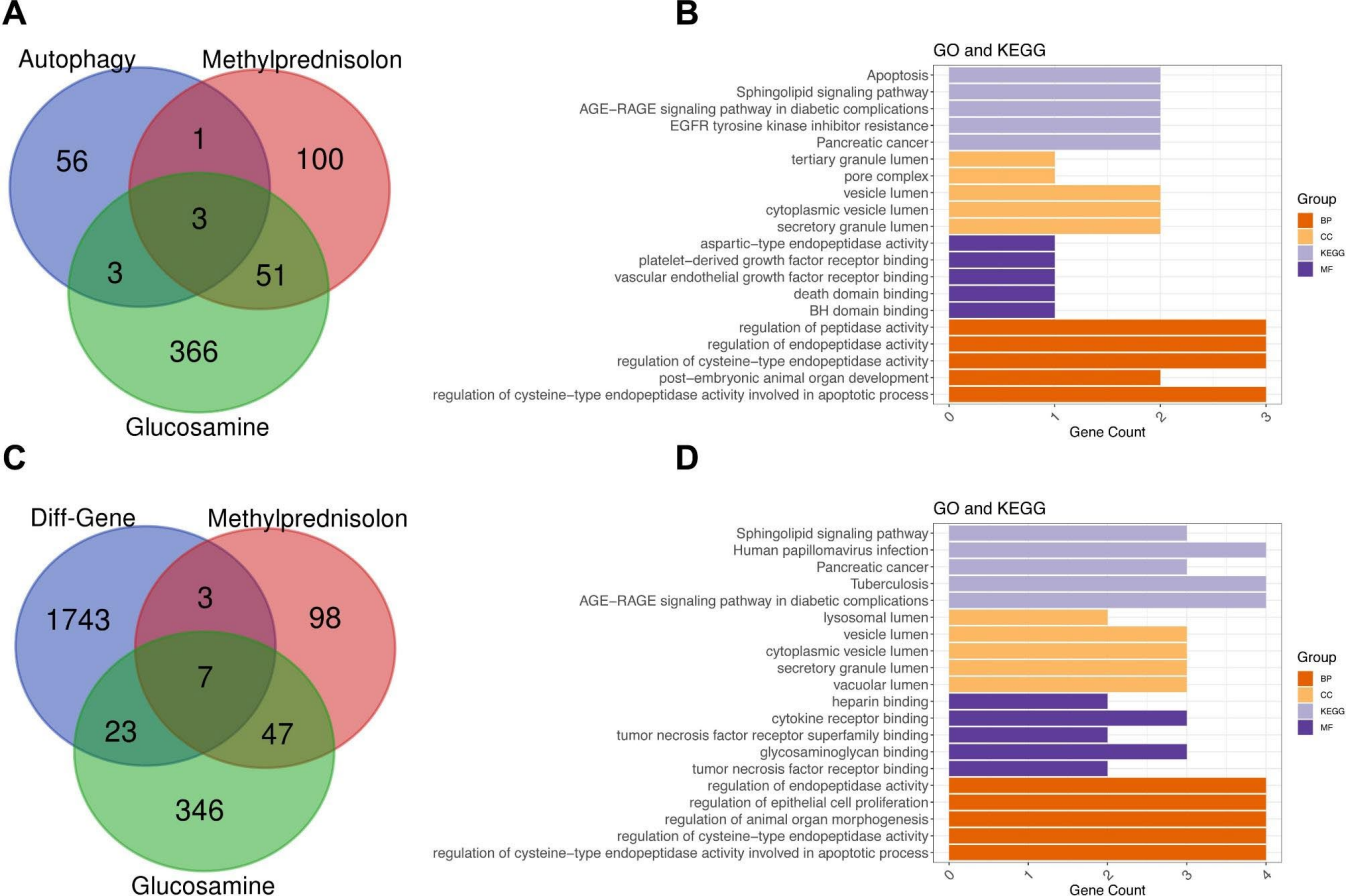
Target genes of methylprednisolone and glucosamine. **(A)** Venn diagram of autophagy-differentially expressed genes (DEGs) and target genes of methylprednisolone and glucosamine **(B)** Enrichment analysis of autophagy-related target genes of methylprednisolone and glucosamine **(C)** Venn diagram of differentially expressed genes (DEGs) and target genes of methylprednisolone and glucosamine **(D) **Enrichment analysis of target DEGs of methylprednisolone and glucosamine

The CLUE website was used to search for drugs related to the five key IVDD-autophagy genes, and 84 significantly related drugs were screened (z-score > 2, P < 0.05). STAT3 was significantly associated with niclosamide, sorafenib, and gemcitabine (median confidence = 0.400, Fig. 8C). The CTD database was used to predict the target genes of methylprednisolone and glucosamine, which intersected with the 63 autophagy-DEGs, and three common autophagy-related target genes of methylprednisolone and glucosamine were obtained (Fig. 9A). The enrichment analysis of these three autophagy-related target genes of methylprednisolone and glucosamine showed that they were significantly enriched in apoptosis, sphingolipid signaling pathway, AGE-RAGE signaling pathway in diabetic complications, and EGFR tyrosine kinase inhibitor resistance (Fig. 9B). Moreover, the predicted target genes of methylprednisolone and glucosamine intersected with 1,776 DEGs, and 7 target DEGs of methylprednisolone and glucosamine were obtained (Fig. 9C). Enrichment analysis showed that the seven target DEGs of methylprednisolone and glucosamine were involved in the sphingolipid signaling pathway, human papillomavirus infection, pancreatic cancer, and tuberculosis (Fig. 9D).

## Discussion

In this study, a diagnostic model was constructed based on five key autophagy-related genes: *PHF23, RAB24, STAT3, TOMM5*, and *DNAJB9*. *PHF23* was recently identified as an autophagy inhibitor, and a previous study demonstrated that *PHF23* inhibition has therapeutic potential in degenerative joint diseases [32]. Hai et al. indicated that *RAB24* might participate in the development of IVDD by triggering numerous immune-associated pathways [33]. Suzuki et al. found that the IL-6/JAK/STAT3 pathway is involved in the pathogenesis of IVDD [34]. *TOMM5* and *DNAJB9* have been reported to be involved in the development of cancer, type 2 diabetes mellitus, and obesity [35–39]; however, few studies of *TOMM5* and *DNAJB9* on IVDD have been reported. Therefore, the functions of *TOMM5* and *DNAJB9* in IVDD should be studied further. In addition, receiver operating characteristic curve was constructed to estimate the predictive ability of the diagnostic model, and AUC values of the model in the training and validation datasets were 0.907 and 0.984, respectively. These indicate that the performance of the diagnostic model is credible. Moreover, enrichment analysis showed that the five key IVDD-autophagy genes in the diagnostic model were significantly enriched in radial glial cell differentiation, regulation of autophagy, and regulation of autophagosome maturation. Published studies have confirmed that autophagy markers exist in IVD tissue, and in vitro, disc cells regulate autophagy in response to cellular stressors [19]. Thus, these five key IVDD-autophagy genes may play a role in IVDD through these biological processes.

Currently, some miRNAs are known to be involved in various pathological processes of IVDD [40, 41]. Zhao et al. showed that miR-19b-3p relieves IVDD by modulating the PTEN/PI3K/Akt/mTOR signaling pathway [42]. Gao et al. found that N6-methyladenosine-induced miR-143-3p promotes IVDD by regulating SOX5 [43]. Wang et al. reported that miRNA-140-3p alleviates IVDD via the KLF5/N-cadherin/MDM2/Slug axis [44]. Thus, in this study, the common miRNAs of five key autophagy-related genes in the diagnostic model were screened, and 16 common miRNAs were identified, including has-miR-8085, has-miR-198, has-miR-6865-5p, and has-miR-6879-5p. Besides, three TFs of the five key IVDD-autophagy genes in the diagnostic model were predicted, including MA0098.1. ETS1, MA1536.1.NR2C2, and MA0719.1.RHOXF1. Thus, it is suggested that these 16 miRNAs and 3 TFs may be involved in the pathogenesis of IVDD by targeting the five key autophagy-related genes.

In total, 84 significantly related drugs of the five key IVDD-autophagy genes were screened. In addition, STAT3 expression was significantly associated with niclosamide, sorafenib, and gemcitabine. A previous study has reported that inflammation is an important factor in the onset and progression of disc degeneration [45]. Li et al. revealed that sorafenib restrains lipopolysaccharide/endotoxin-induced inflammation by regulating Lyn-MAPK-NF-kB/AP-1 pathway and TLR4 expression [46]. Therefore, these 84 drugs can be used as therapeutic agents for IVDD. It has also been reported that methylprednisolone and glucosamine used in the treatment can significantly alleviate lower back and leg pain caused by IVDD and improve spinal cord function [47, 48]. Thus, the target genes of methylprednisolone and glucosamine were predicted to intersect with the 63 autophagy-DEGs, and three common autophagy-related target genes of methylprednisolone and glucosamine were identified, including *CTSD*, *VEGFA*, and *BAX*. Teixeira et al. indicated that *CTSD* modulates the formation of the terminal complement complex in cultured human disc tissues [49]. Feng et al. suggested that Bushen Huoxue decoction intervenes in IVDD through *VEGF-A* [50]. Feng et al. showed that high glucose induces the ChREBP/p300 transcriptional complex to activate the proapoptotic genes, *PUMA* and *BAX*, to contribute to IVDD [51]. Moreover, enrichment analysis showed that these three common autophagy-related target genes of methylprednisolone and glucosamine were significantly involved in apoptosis, sphingolipid signaling, and AGE-RAGE signaling in diabetic complications. Dysregulation of apoptosis has been reported to be involved in the development of degenerative diseases, such as osteoarthritis [52]. Numerous studies have shown that inflammation alters the microenvironment of nucleus pulposus cells, induces apoptosis, and ultimately leads to IVDD [45, 53]. Excessive sphingolipid synthesis can cause degenerative diseases, such as childhood amyotrophic lateral sclerosis [54]. Xia et al. used transcriptome sequencing to identify new therapeutic targets for IVDD and found that DEGs, between the IVDD and non-IVDD groups, were enriched in the AGE-RAGE signaling pathway in diabetic complications [55]. These results support those of the present study, suggesting that methylprednisolone and glucosamine might exert therapeutic effects in IVDD by targeting *CTSD*, *VEGFA*, and *BAX* through apoptosis, sphingolipid signaling pathway, and AGE-RAGE signaling pathway in diabetic complications. However, further in-depth studies are required to confirm these findings.

Nevertheless, this study had some limitations. First, the data were downloaded from public databases; other large-sample datasets are required to validate the results of this study. Second, the five key IVDD-autophagy genes, potential drugs, and target genes, and the molecular mechanisms of methylprednisolone and glucosamine identified in this study should be examined further in other cohorts and in vivo and in vitro experiments. Third, CIBERSORT algorithm was the only one used to estimate the fractions of immune cells infiltration between IVDD and control samples; therefore, flow cytometry should be performed to further validate the reliability of the results.

## Conclusion

In summary, this study developed a reliable autophagy-related diagnostic model that is strongly related to the immune microenvironment of IVD and offers insights into latent therapeutic targets for patients with IVDD. Autophagy-related genes, containing *PHF23*, *RAB24*, *STAT3*, *TOMM5*, and *DNAJB9*, may participate in pathogenesis of IVDD. In addition, methylprednisolone and glucosamine may exert therapeutic effects on IVDD by targeting *CTSD*, *VEGFA*, and *BAX* through apoptosis, sphingolipid signaling pathway, AGE-RAGE signaling pathway in diabetic complications.

## Data Availability

This study analyses publicly available datasets. These data can be found at GSE150408 and GSE124272 (https://www.ncbi.nlm.nih.gov/geo/). All data generated in this study is available from the corresponding author upon reasonable request.
